# 3′ UTR G-quadruplexes regulate miRNA binding

**DOI:** 10.1261/rna.060962.117

**Published:** 2017-08

**Authors:** Samuel Rouleau, Jean-Pierre Sehi Glouzon, Andrea Brumwell, Martin Bisaillon, Jean-Pierre Perreault

**Affiliations:** Département de Biochimie, Pavillon de Recherche Appliquée sur le Cancer, Université de Sherbrooke, Sherbrooke (Québec), Canada J1E 4K8

**Keywords:** RNA G-quadruplex, microRNA, FADS2, mir331-3p

## Abstract

MicroRNAs (miRNAs) are small noncoding RNAs that repress the translation of their target genes. It has previously been shown that a target's availability to miRNA can be affected by its structure. G-quadruplexes (G4) are noncanonical structures adopted by G-rich nucleic acids that have been shown to have multiple biological functions. In this study, whether or not G4 structures’ presence in the 3′ UTRs of mRNAs can hinder miRNA binding was investigated. Putative G4 overlapping with predicted miRNAs’ binding sites was searched for, and 44,294 hits were found in humans. The FADS2 mRNA/mir331-3p pair was selected as a model example. In-line probing and G4-specific fluorescent ligand experiments binding were performed and confirmed the presence of a G4 near the predicted miRNA binding site. Subsequent luciferase assays showed that the presence of the G4 prevents the binding of mir331-3p in cellulo. Together, these results served as proof of concept that a G4 structure present in a 3′ UTR sequence should be taken into consideration when predicting miRNA binding sites.

## INTRODUCTION

MicroRNAs (miRNAs) are small (≈22 nucleotides [nt]) noncoding RNAs that regulate their target genes’ expression (Bartel 2009). In mammals, miRNAs generally recognize their target mRNA by imperfect base-pairing in the 3′ UTR region, although perfect base-pairing is thought to be primordial in the seed region of the miRNA, that is to say in nucleotide positions 2–8 (Chekulaeva and Filipowicz 2009). MiRNA binding usually leads to translational repression and/or an accelerated decay of the target mRNA (Krol et al. 2010). Since miRNAs bind with imperfect complementarity, one miRNA can regulate dozens or even hundreds of different targets. MiRNAs are thus master regulators, and an imbalance in their own expression level can lead to various phenotypes, including health problems, such as cardiovascular diseases (Small and Olson 2011), neurodegenerative diseases (Abe and Bonini 2013), and cancer (Farazi et al. 2013). Consequently, they are considered important therapeutic targets (Hayes et al. 2014; Taylor and Schiemann 2014).

G-quadruplexes (G4) are noncanonical structures adopted by guanine-rich DNA or RNA sequences (Huppert 2008). Recently, multiple teams used structure-specific antibodies to observe the formation of both DNA and RNA G4 in cells (Biffi et al. 2013, 2014; Henderson et al. 2014). RNA G4 are more stable and display less topological diversity than their DNA counterparts. They are also more likely to fold because the single-stranded RNA lacks a complementary strand, thus favoring intramolecular G4 base-pairing. Several biological functions for RNA G4 have been reported, including roles in translational repression, RNA splicing, mRNA polyadenylation, and mRNA localization (Millevoi et al. 2009; Agarwala et al. 2015).

It has been shown that the target's secondary structure can greatly influence miRNA efficiency (Liu et al. 2014). Indeed, highly structured regions are less accessible to miRNA binding. Long et al. (2007) have shown the necessity of target accessibility by manipulating the binding between the *C. elegans* miRNA let-7 and one of its targets, specifically the lin-41 mRNA. The region spacing two imperfectly complementary miRNA binding sites was mutated to reduce the target's accessibility, without changing the sequence of the miRNA binding site itself. When the site was rendered inaccessible, the repression of lin-41 by let-7 was found to be minimal to absent. Therefore, they concluded that the structural accessibility of the target was essential to miRNA binding. However, the potential influence of G4 on miRNA remains as yet a largely unaddressed question.

In one of the only examples published to date, Woo et al. (2013) showed that a G4 present in the 3′ UTR of the CSF-1 gene is bound by the nucleolin protein, a process that enhances the miRNA-mediated mRNA decay. In another report, Booy et al. (2013) showed that RHAU, a G4 helicase also known as DHX36, negatively regulates the PITX1 gene by binding to a G4 located in the 3′ UTR of its mRNA. These authors also showed that this regulation was both Dicer- and Ago2-dependent. However, the exact link between the miRNAs and the G4 was not clearly defined. Recently, Stefanovic et al. (2015) have shown that the PSD-95 3′ UTR contains multiple potential G4 structures, the folding of which might regulate mir-125a binding. This hypothesis was based solely on in vitro data, and so far, there are no reports of either in cellulo or in vivo experiments confirming this mechanism.

**FIGURE 1.**
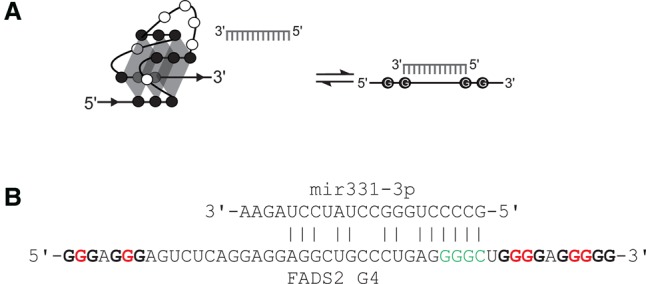
The presence of a G-quadruplex prevents miRNA binding. (*A*) Schematic illustration of the equilibrium between G4 folding and miRNA binding. (*B*) The putative FADS2 G4 sequence and the predicted mir331-3p binding site. Nucleotides mutated in the G/A mutant are shown in red and nucleotides mutated in the target mutant are shown in green.

The present study aims to characterize the influence of the G4 presence within the mRNA on miRNA binding. Specifically, it was hypothesized that G4 formation near the miRNA binding site may in fact prevent miRNA binding (Fig. 1A). Several miRNA target predictors were used to find putative miRNA binding sites that overlapped with predicted G4 structures. Over 44,000 hits were found in human (i.e., specifically 44,294 hits). The fatty acid desaturase 2 (FADS2) mRNA, predicted to be a target of mir331-3p, was selected as a prime example. FADS2's 3′ UTR was shown to contain a sequence that was able to fold into a G4 in vitro, while luciferase reporter assays showed that the presence of the G4 had a significant impact on miRNA binding in cells.

## RESULTS AND DISCUSSION

### Putative miRNA binding sites overlap with predicted G4 structures

Initially, putative G4 overlapping with predicted miRNA binding sites located in the 3′ UTRs of human genes were searched for (see Materials and Methods for details). It has previously been shown that G4s with a long central loop can fold correctly and exert a biological function in cells (Jodoin et al. 2014). Therefore, sequences corresponding to the motif G_*X*_N_1_G_*X*_N_*Y*_G_*X*_N_1_G_*X*_, where *X* ≥ 3 and 1 ≤ *Y* ≤ 120, were investigated. G4 of this length were chosen so that they can either roughly overlap, or entirely contain, the miRNA binding sites. A total of 2282 putative G4 were found in 1112 genes (Supplemental File 1). This included 1233 (54%) G4 in 611 genes that were predicted to overlap with at least one miRNA binding site. On the other hand, 521 unique miRNAs were predicted to bind to at least one G4. Overall, 44,294 potential G4/miRNA binding site pairs were found (Supplemental File 2). Among these, 13,361 (30%) were predicted to bind exclusively in the central loop while the rest were predicted to bind at least one stretch of guanines. The results are summarized in Supplemental Table 1. A gene ontology analysis (see Materials and Methods sections for details) was performed on the target 611 genes containing a putative G4 predicted to be bound by at least one miRNA. Results are shown in Supplemental File 3. In brief, a few biological functions were shown to be moderately enriched. The greatest enrichment was for positive regulation of mitochondrial outer membrane permeabilization involved in an apoptotic signaling pathway (7.5-fold). Interestingly, 24 of the 85 GO terms found to be enriched at least twofold, and 13 of the 20 most enriched GO terms were related to brain development, neurogenesis, and neural function. Several studies have shown that 3′ UTR G4 can serve as localization signal in neurons (Subramanian et al. 2011; Schofield et al. 2015; Ishiguro et al. 2016).

The fatty acid desaturase 2 (FADS2) mRNA, a cancer-related gene, was chosen as a prime example. Also known as delta-6-desaturase, this enzyme introduces double bonds into defined carbons of fatty acyl chains. It is the rate-limiting metabolic enzyme in the conversions of linoleic acid into arachidonic acid (AA) and of α-linolenic acid into eicosapentanoic acid (EPA) (Park et al. 2015). Both AA and EPA are involved in the inflammatory response (Vaittinen et al. 2016). FADS2 activity is known to be increased in breast cancer; therefore, its inhibition is a potential treatment method (Lane et al. 2003; Pender-Cudlip et al. 2013). The 3′ UTR of the FADS2 mRNA is 1664 nt long and possesses a potential G4 sequence located between positions 1207 and 1252. The probability of the formation of this G4 is relatively high since it has a 4.44 value according to the cGcC scoring system (see Beaudoin et al. 2014 for details on this scoring system). According to the three softwares used (see Materials and Methods section for details), this 3′ UTR region possesses a putative miRNA binding site for mir331-3p in positions 1222 to 1244, which overlaps with the putative G4 (Fig. 1B). In this case, it could be possible that the G4 structure impairs the binding of mir331-3p.

### The selected candidate folds into a G4 structure in vitro

In-line probing was used to verify if the FADS2 3′ UTR can fold into a G4 in vitro. This technique is based on an RNA's ability to self-cleave spontaneously via a nucleophilic attack of the ribose's 2′-hydroxyl group on the backbone's phosphodiester bond. The more flexible a given nucleotide is, the greater the amount of cleavage that can be detected at that particular position by denaturing polyacrylamide gel electrophoresis. The folding of a G4 structure confers added flexibility to both the loop nucleotides and to those adjacent to the G tracts, thus rendering this stretch of nucleotides more susceptible to in-line attack. Therefore, by comparing the cleavage pattern obtained in the presence of Li^+^, which does not support G4 folding, with that obtained in the presence of K^+^, which does, one can assess G4 formation (Beaudoin et al. 2013).

**FIGURE 2.**
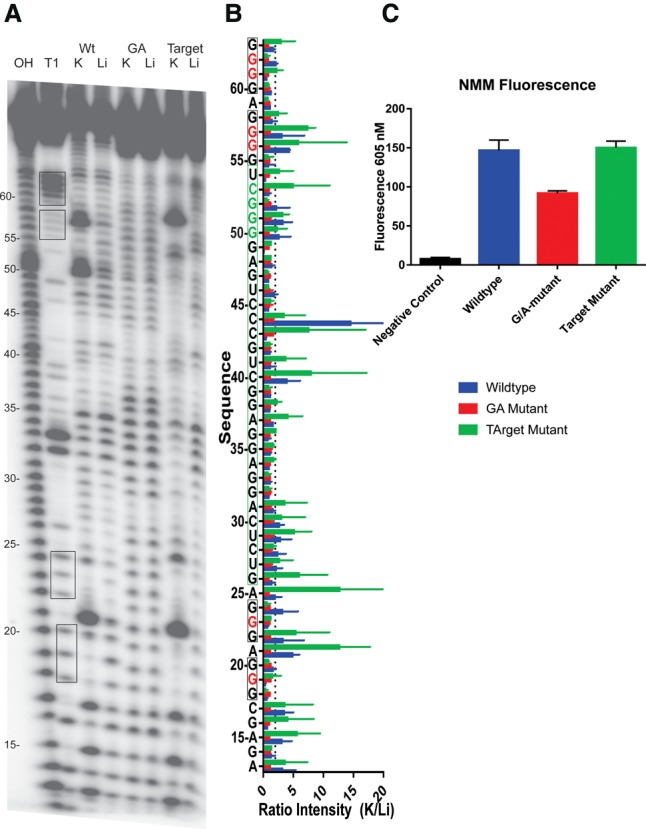
The FADS2 3′ UTR G-quadruplex folds in vitro. (*A*) In-line probing gel of the FADS2 G4. The alkaline digestion (all nucleotides), the T1 digestion (only the guanines), and the in-line probing performed in the presence of K^+^ (favors G4) or Li^+^ (does not favor G4) for the wild-type, G/A mutant, and the target mutant of FADS2 are shown. The guanines involved in the G4 are boxed in the T1 lane. (*B*) Quantification of the in-line probing experiments. Each band was quantified using the SAFA software, and a K^+^/Li^+^ ratio was calculated for each nucleotide. A ratio >2 for the nucleotides located in the loops indicates G4 folding. The means and standard deviations were calculated from two different experiments. The Guanines involved in the G4 are boxed in black, and the nucleotides mutated in the G/A mutant are shown in red. The mir331-3p-binding site is boxed in green and the nucleotides mutated in the target mutant are shown in green. (*C*) *N*-methyl mesoporphyrin IX (NMM) fluorescence assay. The NMM fluorescence in the presence of FADS2 and the G/A and the target mutants are shown. The means and standard deviations were calculated from two different experiments.

The predicted G4 sequence, as well as ≈15 nt flanking the G4 (in order to mimic the genomic context), was in vitro transcribed and radiolabeled at the 5′ end (see Supplemental Table 2 for the oligonucleotide sequences). In-line probing was performed on the wild-type sequence, a G/A mutant, and a target mutant (see Fig. 2B). In the G/A mutant, the central guanines of a few G tracts were substituted for adenines in order to prevent the G4 formation without changing the miRNA binding site (see the red nucleotides in Figs. 1, 2B). In the target mutant, a few nucleotides of the miRNA's seed region's predicted binding site were mutated in order to prevent miRNA binding without affecting the predicted G4 formation (see the green nucleotides in Figs. 1, 2B). The resulting mixtures were analyzed on a denaturing (7 M urea) 10% polyacrylamide gel. As expected, the in-line cleavage patterns revealed G4 formation in both the wild-type and the target mutant sequences, as nucleotides located in the loops were more reactive in the presence of K^+^ than in the presence of Li^+^ (Fig. 2A,B). This was not the case for the G/A mutant sequence, which showed similar cleavage patterns in the presence of K^+^ and Li^+^. It should be noted that the miRNA binding site contains a stretch of four guanines that could potentially be implicated in alternative G4 conformation. This could explain the small differences in cleavage patterns between the wild-type and target mutant. However, regardless of the guanines involved in the G4 folding, the structure would still be able to impair miRNA binding.

As a complementary method, *N*-methyl mesoporphyrin IX (NMM) staining assays were performed. NMM is a parallel G4-specific ligand that is fluorescent only when bound to a G4 (Sabharwal et al. 2014). Both FADS2 wild-type and target sequences showed high fluorescence levels as compared to that of either the G/A sequence or NMM alone (negative control) (Fig. 2C). The low fluorescence of the G/A mutant could be explained by the presence of a less stable two-tiered G4 that only folds in the presence of the NMM. These results were in accordance with the in-line experiments, confirming that the potential G4 sequence of the FADS2 3′ UTR indeed folds into a stable G4 structure.

### G4 formation effect on miRNA binding

**FIGURE 3.**
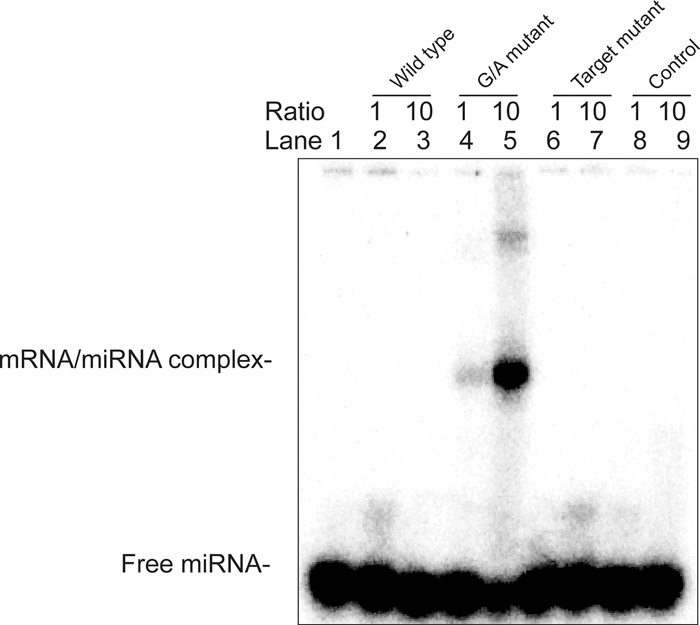
The FADS2 G-quadruplex prevents mir331-3p binding in vitro. Native polyacrylamide gel electrophoresis of radiolabeled mir331-3p by itself (0.1 µM) and with different amounts (either 0.1 or 1 µM) of either FADS2 wild-type, G/A mutant, target mutant, or an unrelated RNA control are shown.

In order to see the impact of G4 formation on miRNA binding, gel shift experiments were performed. Radiolabeled mir331-3p RNA molecules were incubated with the same FADS2 transcripts that were used in the in-line probing assays and with an unrelated RNA that was used as a nonspecific control. The RNAs were then run on a native polyacrylamide gel in order to see if the interactions with the target would cause a shift in the mir331's migration (Fig. 3). Only the G/A mutant sequence (lanes 4 and 5) induced a shift in migration, indicating that the presence of the G4 within the target prevents miRNA binding. Furthermore, increasing the target RNA concentration led to a larger proportion of shifted miRNA (lane 5 as compared to lane 4).

**FIGURE 4.**
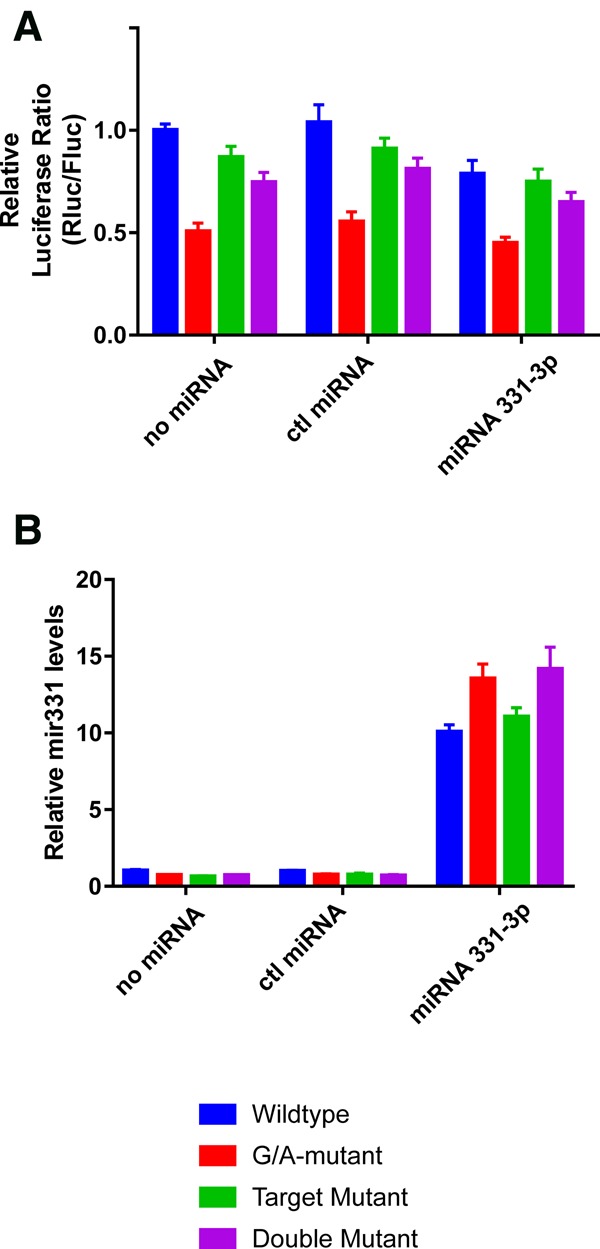
The FADS2 G-quadruplex prevents mir331-3p binding in cells. (*A*) The luciferase activities of the different FADS2 constructs (wild-type, G/A mutant, target mutant, and double mutant) when transfected into HEK 293 cells either alone, with a control synthetic miRNA, or with a synthetic mir331-3p. (*B*) RT-qPCR measured levels of mir331-3p are shown under the same conditions as in *A*.

In order to see if the G4 can impair miRNA binding in a more biologically relevant context, luciferase assays were performed. The full FADS2 3′ UTR sequence, as well as G/A mutant and target mutant sequence versions, was inserted downstream from a luciferase reporter. As an additional control, a double mutant, which harbors mutations in the G4 forming sequence as well as in the miRNA binding site, was also used. The luciferase plasmids were transfected into HEK 293 cells either alone, with a synthetic control nontargeting miRNA, or with a synthetic mir331-3p. The results are shown in Figure 4A. As expected, mir331 concomitant transfection only led to a modest decrease in luciferase activity for the wild-type construct, as the G4 prevented miRNA binding. Mutation of the G4 led to a twofold decrease in luciferase activity, even in the no miRNA and the control miRNA transfected cells. This can be explained by the presence of endogenously produced mir331-3p, which could be detected by RT-qPCR (Fig. 4B). To confirm that endogenous miRNA could regulate luciferase expression, a mir331-3p inhibitor was used (see Supplemental Fig. 4). While the inhibitor led to a modest increase of the wild-type luciferase expression (33% increase at the highest concentration tested), it led to a bigger increase of the G/A mutant expression (66% increase at the highest concentration tested). Still, the luciferase activity was at its lowest with the G/A mutant and in the presence of the synthetic mir331-3p, as expected. The target mutant construct had luciferase activity similar to those of the wild-type, regardless of which synthetic miRNA was transfected, further supporting the notion that the G4 prevents miRNA binding. Indeed, if miRNA binding was possible, the mutation of the binding site would have led to an increase in luciferase activity, which was not observed. Interestingly, when both the G4 and the miRNA binding sites were mutated, the luciferase activity was almost rescued to a wild-type level. This indicates that the reduced luciferase activity observed in the case of the G/A mutant is largely due to mir331-3p binding, as most of this effect is lost in the double mutant. The reduced activity in the double mutant, as compared to that of the wild-type, might be explained by the binding of different endogenously produced miRNAs or by an miRNA independent effect specific to the G4. More importantly, taken together, these results clearly demonstrate that the G4 present in the FADS2 3′ UTR prevents mir331-3p binding.

### Are there other G4 regulating miRNA binding?

In order to see if other G4 located in the 3′ UTRs of different genes could act in a similar manner, a few more potential G4/miRNA pairs were selected for further testing: i.e., the ELK1/mir423-5p, HDGF/mir92a-3p, THRB/mir204-5p, and Wnt3a/mir132-3p pairs. Although all of the predicted G4 folded in vitro (Supplemental Figs. 1, 2), luciferase assays showed no evidence of any G4 effect on miRNA binding (Supplemental Fig. 3). This result could be explained by multiple factors. First, the G4 might not be able to correctly fold in cells in the context of the full mRNA, as it has been observed for other predicted G4 (Beaudoin and Perreault 2010; Beaudoin et al. 2014). Another possibility is that these genes are not bona fide targets of their respective miRNAs, and that using different miRNAs could lead to better results. Finally, since the miRNAs and their targets are intricate parts of complex regulatory networks, which differ greatly among different tissues and situations, the choice of cell line is crucial in order for the results to be as biologically relevant as possible. Therefore, whenever possible, in addition to the HEK 293 cells that were used for all of the genes tested here, cell lines derived from the tissues in which the candidates are known to be associated with specific types of cancer were also used. Hence, the Wnt3a plasmids were transfected into HCT 116 cells, a colorectal cancer-derived cell line, and the HDGF plasmids were transfected into Huh7 cells, a hepatoma-derived cell line. For both THRB and ELK1, only the HEK293 cell line was used. In all cases, the inhibition of the miRNA binding resulting from the formation of the G4 structure could not be observed under the conditions tested here. It is possible that further testing in other cell lines could lead to positive results.

Although only 20% of the G4 tested were shown to prevent miRNA binding, the very high number of potential G4/miRNA pairs (i.e., 44,294) suggests that a significant number of miRNA binding sites could be influenced by the presence of a G4. Furthermore, in this study, only G4 possessing a long loop 2 were investigated, but it has been shown that G4 possessing either a long loop 1 or 3 can also fold and have an impact on gene expression (Bolduc et al. 2016). Moreover, G4 adjacent to, but not overlapping, an miRNA binding site could also have either a positive or a negative effect on miRNA binding by changing the global structure of the mRNA (Stefanovic et al. 2015). That said, confirmation of this requires further experiments that are beyond the scope of this study.

### Conclusion

In summary, it was shown that the G4 present in the 3′ UTR of the FADS2 mRNA prevents the binding of mir331-3p. Therefore, G4 present in the target should be taken into account when predicting miRNA binding sites. G4 folding is a dynamic process. Recently, Guo and Bartel (2016) reported that although tens of thousands of transcribed RNA sequences have the potential to fold into a G4 in vitro, only a small fraction of these actually adopt this structure in cellulo. It should be noted, however, that cellular stresses and the expression levels of G4 helicases and other RNA binding proteins could modulate the G4 folding equilibrium. This could add another layer of complexity to the miRNA mediated fine-tuning of gene expression.

## MATERIALS AND METHODS

### Bioinformatic analysis

The computational approach used here to identify human G4 that can potentially impede miRNA binding was designed as follows: first, genes whose 3′ UTR included the motif G_*X*_N_1_G_*X*_N_*Y*_G_*X*_N_1_G_*X*_ where *X* ≥ 3 and 1 ≤ *Y* ≤ 120 were selected (Supplemental File 1). Second, three different miRNA target prediction softwares (miRANDA [Enright et al. 2003], RNAhybrid [Krüger and Rehmsmeier 2006], and Pita [Kertesz et al. 2007]) were used to find miRNA binding sites overlapping with the putative G4. A mirPearl script (Porto et al. 2015) was used to keep only the miRNAs that were predicted to bind on the same G4 by the three softwares (Supplemental File 2).

### Gene Ontology analysis

The Panther software (Mi et al. 2013) was used to perform the Gene Ontology analysis on all the genes containing at least one G4 predicted to be bound by at least one miRNA. The parameters were set as follows: Analysis Type: PANTHER Overrepresentation Test (release 20160715), Annotation Version and Release Date: GO Ontology database Released 2017-02-28, Reference List: Homo sapiens (all genes in the database), Annotation Data Set: GO biological process complete, Bonferroni correction: enabled.

### RNA synthesis

All RNA molecules used both for the in-line probing and the gel shift assays were synthesized by in vitro transcription using T7 RNA polymerase as described previously (Beaudoin et al. 2013). Briefly, two overlapping oligonucleotides (2 µM each, see Supplemental Table 2 for the sequences) were annealed, and double-stranded DNA was obtained by filling in the gaps using purified *Pfu* DNA polymerase in the presence of 5% dimethyl sulfoxide (DMSO). The double-stranded DNA was then ethanol-precipitated. The resulting DNA templates contained the T7 RNA promoter sequence followed by the G4 sequence and 15 flanking nucleotides. After dissolution of the polymerase chain reaction (PCR) product in water to a final concentration of 2–5 mM, the transcription reactions were performed for 2 h at 37°C in the presence of purified T7 RNA polymerase (10 μg) and pyrophosphatase (0.01 U, Roche Diagnostics) in a buffer containing 80 mM HEPES–KOH (pH 7.5), 24 mM MgCl_2_, 2 mM spermidine, 40 mM DTT, and 5 mM of each NTP in a final volume of 100 μL. Upon completion, the reaction mixtures were treated with DNase RQ1 (Promega) at 37°C for 15 min, and the RNA was then purified by phenol/chloroform extraction and ethanol precipitation. The resulting pellets were dissolved in a 1:2 ratio of water to loading buffer (95% formamide, 10 mM EDTA [pH 8.0], and 0.025% bromophenol blue). These samples were then fractionated through 8% denaturing polyacrylamide gels (PAGE, 19:1 ratio of acrylamide to bisacrylamide) in buffer containing 45 mM Tris–borate (pH 7.5), 8 M urea, and 2 mM EDTA. The reaction products were visualized by ultraviolet (UV) shadowing. The bands corresponding to the correct sizes were cut out, and the transcripts were eluted overnight in elution buffer (500 mM ammonium acetate, 10 mM EDTA, and 0.1% SDS). The eluted transcripts were then ethanol-precipitated, dried, and dissolved in 50 µL of water. The RNA was then quantified by absorbance at 260 nm.

### RNA labeling

Purified transcripts (50 pmol) were dephosphorylated for 1 h at 37°C in a solution containing 1 U of Antarctic phosphatase (New England Biolabs), 50 mM bis-propane (pH 6.0), 1 mM MgCl_2_, 0.1 mM ZnCl_2_, and RNase OUT (20 U, Invitrogen) in a final volume of 10 µL. The enzyme was then inactivated by incubating for 5 min at 65°C. Dephosphorylated transcripts (5 pmol) were 5′-end-radiolabeled using 3 U of T4 polynucleotide kinase (Promega) for 1 h at 37°C in the presence of 3.2 pmol of [α-^32^P]ATP (6000 Ci/mmol; New England Nuclear) in a final volume of 10 µL. The reactions were stopped by the addition of 20 µL of formamide dye buffer (95% formamide, 10 mM EDTA, and 0.025% bromophenol blue), and the RNA was then purified by 10% polyacrylamide gel electrophoresis. The bands of the correct sizes containing the 5′-end-labeled RNAs were excised and the RNA recovered as described above, except that the detection was performed by autoradiography.

### In-line probing

Trace amounts of labeled RNA (≈50 000 cpm) were heated at 70°C for 5 min and then were slow-cooled to room temperature over 1 h in buffer containing 50 mM Tris–HCl (pH 7.5), and 100 mM of either LiCl or KCl in a final volume of 10 µL. Following this incubation, the final volume of each sample was adjusted to 20 µL such that the final concentrations were 50 mM Tris–HCl (pH 7.5), 20 mM MgCl_2_, and 100 mM of either LiCl or KCl. The reactions were then incubated for 40 h at room temperature. The RNA was then ethanol-precipitated and dissolved in 20 µL of formamide dye loading buffer (95% formamide, 10 mM EDTA, and 0.025% bromophenol blue). For alkaline hydrolysis, 5′-end-labeled RNA (50,000 cpm) was dissolved in 5 µL of water, 1 µL of 1 N NaOH was added, and the reactions were incubated for 1 min at room temperature prior to being quenched by the addition of 3 µL of 1 M Tris–HCl (pH 7.5). The RNA molecules were then ethanol-precipitated and dissolved in 20 µL of formamide dye loading buffer. RNase T1 ladders were prepared using 50,000 cpm of 5′-end-labeled RNA dissolved in 10 µL of buffer containing 20 mM Tris–HCl (pH 7.5), 10 mM MgCl_2_, and 100 mM LiCl. The mixtures were incubated for 2 min at 37°C in the presence of 0.6 U of RNase T1 (Roche Diagnostic), and the reactions were then quenched by the addition of 20 µL of formamide dye loading buffer. For practical reasons, Alkaline digestion and T1 ladder were prepared with G/A or target mutant. The radioactivity of the in-line probing samples and of both of the ladders were calculated, and equal amounts (in terms of counts per minute) of all conditions and ladders of each candidate were fractionated on denaturing (8 M urea) 10% polyacrylamide gels. The RNA was then visualized by exposure to a phosphorscreen (GE Healthcare) using a Typhoon Trio instrument (GE Healthcare). The SAFA (Semi Automated Footprinting Analysis) software was used to quantify each band. The intensity of the band incubated in the presence of KCl was then divided by the intensity of the corresponding band incubated in the presence of LiCl. The histograms show the mean results of two separate experiments.

### NMM fluorescence

NMM, a stain that is fluorescent only in the presence of parallel G4s, was used as a supplementary method of confirming the presence of the predicted G4s. The protocol of Booy et al. (2012) for in vitro G4 staining was modified, so that the assays could be performed in 1.5 mL Eppendorf tubes. In vitro transcribed RNA (1000 pmol) was incubated with 100 µL of 1× K Res Helicase Buffer (100 mM KCl, 10 mM NaCl, 3 mM MgCl_2_, 50 mM Tris–Acetate [pH 7.8], 70 mM glycine, 0.012% BSA, and 10% glycerol) for 5 min at 70°C, followed by slow-cooling to room temperature. Once at room temperature, 25 µM of NMM was added, and samples were incubated for 30 min at room temperature. The fluorescence of the samples was then measured with an excitation wavelength of 399 nm and an emission wavelength of 550–650 nm using a Hitachi F-2500 fluorescence spectrophotometer.

### Gel shift assays

MiRNAs were ordered as RNA oligonucleotides from Integrated DNA Technologies (see Supplemental Table 2 for the sequences) and were radiolabeled as described above. Trace amounts (≈5000 cpm) of labeled miRNA were added to 1 pmol of cold miRNA. Either 1 or 10 pmol of either wild-type or mutant target sequences (the same ones that were used in the in-line probing experiments described above) were added to the mix in 100 mM Tris–HCl (pH 7.0), 100 mM KCl buffer to a final volume of 10 µL. The THRB wild-type sequence was used as a nonspecific control. The samples were heated at 65°C for 5 min and were then slow-cooled to 37°C in order to favor G4 formation. The samples were then incubated for 30 min at 37°C to favor miRNA binding under cell-like conditions. Lastly, the samples were run on a 10% native polyacrylamide gel for 3–4 h at 4°C. The RNA was then visualized by exposure to a phosphorscreen (GE Healthcare) using a Typhoon Trio instrument (GE Healthcare).

### DNA constructs

With one exception, all plasmids were purchased from Switchgear Genomics pLightSwitch 3′ UTR collection. The THRB partial 3′ UTR (nucleotides from positions 1672 to 4672 from NM_000461) was purchased from Creative Biogene and subcloned into the pLightSwitch vector from Switchgear Genomics using the XhoI and NheI restriction sites. G/A, target, and double mutants were then generated using the Q5 Site-Directed Mutagenesis Kit (New England Biolabs).

### Cell culture and dual luciferase assay

HEK293 and Huh7 cells were cultured in 100 mm petri dishes (Sarstedt) in Dulbecco's modified Eagle medium (DMEM), while HCT116 cells were cultivated in McCoy's medium. All media were supplemented with 10% fetal bovine serum (FBS) and 1 mM sodium pyruvate (all purchased from Wisent). All cells were kept at 37°C in a 5% CO_2_ atmosphere in a humidified incubator.

For transfection, the cells were seeded in 24-well plates (Sarstedt) at 100,000 cells/well. Each well was transfected with 500 ng of pLightSwitch plasmid, 50 ng of pGL3 control plasmid (to assess the transfection efficiency), and 30 pmol of synthetic miRNA (switchgear genomics) using 2 µL of lipofectamine 2000 per well and OPTI-MEM media (Life technologies). At 24 h post-transfection, the cells were lysed, and both the Renilla luciferase (RLuc) and Firefly luciferase (FLuc) activities were measured using the Dual-luciferase Reporter Assay Kit (Promega) according to the manufacturer's protocol using a GloMax 20/20 Luminometer (Promega). For each lysate, the value of the RLuc was divided by that of the FLuc. Each transfection condition was performed in triplicate and was repeated three separate times. For the mir331-3p inhibitor experiment, 0, 20, 30, 40, or 50 nM of inhibitor (IDT) was transfected on the first day using 2 µL of lipofectamine 2000 per well. On the next day, 500 ng of pLightSwitch plasmid and 50 ng of pGL3 control plasmid were transfected using 0.5 µL of lipofectamine 2000 per well. Twenty-four hours after the second transfection, cells were lysed and luciferase assayed as described above.

### Quantitative PCR

Small RNAs were extracted from whole cells using the miRVANA microRNA Isolation Kit (Invitrogen), according to the manufacturer's protocol. RNA integrity and concentration were assessed with a Qubit 2.0 Fluorometer (Invitrogen by Life Technologies) with the Qubit microRNA Assay Kit (molecular probes by Life Technologies). Reverse transcription was performed on 25 ng of RNA with Maxima Enzyme Mix (Maxima First Strand cDNA Synthesis Kit for RT-qPCR [Thermo Scientific]), stem–loop RT specific primer, dNTPs (Roche Diagnostics), and 10 units of RNAseOUT (Invitrogen) following the manufacturer's protocol in a total volume of 10 µL. All forward and reverse primers, as well as Taqman probes, were individually resuspended with 20–100 μM stock solution in Tris–EDTA buffer (IDT). Quantitative PCR (qPCR) reactions were performed in 10 µL in 384 well plates on a CFX-384 thermocycler (BioRad) with 0.2 U of Platinum Taq (5 U/µL Invitrogen), 1.2 µM TaqMan probe, 1.5 µM forward specific primer, 0.7 µM reverse universal primer, 1× Buffer Taq 10×, 200 µM dNTPs, 1.5 mM MgCl_2_, and 3 µL of diluted (1:20) RT reaction. The following cycling conditions were used: 10 min at 95°C; 40 cycles: 15 sec at 95°C, and 60 sec at 60°C. Relative expression levels were calculated using the qBASE framework (Hellemans et al. 2007), and the housekeeping genes used were miR-16, -142, and Let-7a. Primers were designed as described previously (Chen et al. 2005) using the miRBase miRNA database (Griffiths-Jones et al. 2006). Primer validation was evaluated as described elsewhere (Brosseau et al. 2010). In every qPCR run, a no-template control and a no-RT control were performed for each primer pair, and these were consistently negative. All primer sequences are available in Supplemental Table 1.

## SUPPLEMENTAL MATERIAL

Supplemental material is available for this article.

## Supplementary Material

Supplemental Material
