# Supplementary material for: 3′ UTR G-quadruplexes regulate miRNA binding
Source: RNA. 2017 Aug;23(8):1172–9. doi: 10.1261/rna.060962.117 (PMC5513062; doi:10.1261/rna.060962.117)
Supplement: Supplemental Material [file supp_23_8_1172__index.html]

3′ UTR G-quadruplexes regulate miRNA binding — Supplemental Material 

# 3′ UTR G-quadruplexes regulate miRNA binding

## Supplemental Material

- Supplemental\_File\_2\_G4\_overlapping\_with\_miRNA\_binding\_sites.xlsx
- Supplemental\_File\_3\_GO\_analysis.xlsx
- Supplemental\_File\_4\_Instructions\_for\_the\_miRP\_scripts.pdf
- Supplemental\_Material.pdf
- Supplemental\_File\_1\_G4\_in\_the\_3\_UTR\_of\_human\_mRNAs.xlsx
