# Supplementary material for: 3′ UTR G-quadruplexes regulate miRNA binding
Source: RNA. 2017 Aug;23(8):1172–9. doi: 10.1261/rna.060962.117 (PMC5513062; doi:10.1261/rna.060962.117)
Supplement: Supplemental Material [file supp_060962.117_Supplemental_File_4_Instructions_for_the_miRP_scripts.pdf]

miRP

microRNA target prediction auxiliary Perl Scripts – version 1.01

Disclaimer 1
Introduction 2
Scripts interface and installation 2
Getting started 3
Performing a systematic scan of a mRNA sequence 4
miRP-Runner 4
The split folder 5
The results folder 5
- miranda-results-full-records-sorted.csv 6
- miranda-results-seed.csv 6
- hybrid-results-full-records-sorted.csv 6
- hybrid-results-seed.csv 7
- hybrid-results-3end.csv 7
- pita-results-full-records-sorted.csv 7
- pita-results-seed.csv 7
- miRNA-compared.csv 8
- best-mirna.csv 8
Using mirp-runner without a prediction method 8
miRP-miRanda, miRP-Hybrid and miRP-Pita 8
Looking for miRNA that are influenced by SNPs or haplotypes 9
miRP-SNP 9
The results folder 10
- miranda-data1-SNP.csv and miranda-data2-SNP.csv (when comparing mirp-runner or mirp-miranda runs) 10
- hybrid-data1-SNP.csv and hybrid-data2-SNP.csv (when comparing mirp-runner or mirp-hybrid runs) 10
- pita-data1-SNP.csv and pita-data2-SNP.csv (when comparing mirp-runner or mirp-pita runs) 10
- miranda-compared-SNP.csv (when comparing mirp-runner or mirp-miranda runs) 10
- hybrid-compared-SNP.csv (when comparing mirp-runner or mirp-hybrid runs) 11
- pita-compared-SNP.csv (when comparing mirp-runner or mirp-pita runs) 11
Other supporting scripts 12
mirp-csv 12
mirp-fastafilter-file.pl 12
mirp-fastafilter-string.pl 12
mirp-seqsplit.pl 12
Known issues and bug reporting 13
Version history 13
References 13

Disclaimer

THESE SCRIPTS ARE PROVIDED "AS IS" AND ANY EXPRESSED OR IMPLIED WARRANTIES, INCLUDING, BUT NOT LIMITED TO, THE IMPLIED WARRANTIES OF MERCHANTABILITY AND FITNESS FOR A PARTICULAR PURPOSE ARE DISCLAIMED. IN NO EVENT SHALL THE AUTHOR BE LIABLE FOR ANY DIRECT, INDIRECT, INCIDENTAL, SPECIAL, EXEMPLARY, OR CONSEQUENTIAL DAMAGES (INCLUDING, BUT NOT LIMITED TO, PROCUREMENT OF SUBSTITUTE GOODS OR SERVICES; LOSS OF USE, DATA, OR PROFITS; OR BUSINESS INTERRUPTION).

HOWEVER CAUSED AND ON ANY THEORY OF LIABILITY, WHETHER IN CONTRACT, STRICT LIABILITY, OR TORT (INCLUDING NEGLIGENCE OR OTHERWISE) ARISING IN ANY WAY OUT OF THE USE OF THIS SOFTWARE, EVEN IF ADVISED OF THE POSSIBILITY OF SUCH DAMAGE.

Introduction

The miRP package comprises a series of Perl scripts that assist the prediction of miRNA targets by using three different prediction algorithms, miRanda (1), available at http://www.microrna.org/microrna/getDownloads.do, RNAHybrid (2), available at http://bibiserv.techfak.uni-bielefeld.de/rnahybrid/ and PITA (3), available at http://genie.weizmann.ac.il/pubs/mir07/mir07_data.html.

The first two algorithms were designed for calculating the Minimum Free Energy (MFE) of hybridization between a mRNA sequence (the target) and mature miRNAs sequences (the query) based in traditional Watson and Crick base-pairing and the possible structure of these bindings (double-stranded RNA). The third one, PITA, is a parameter-free model that computes the difference between the free energy gained from the formation of the microRNA-target duplex and the energetic cost of unpairing the target to make it accessible to the microRNA.

miRP presents scripts that assist the analyses specially of long mRNA sequences (although short sequences are also acceptable) by fragmenting the mRNA sequence in a series of overlapping subsequences as previously defined by the user. The algorithms are then used to predict the miRNA/mRNA interactions considering each of these subsequences. This step maximizes the ability of these algorithms to predict miRNA/mRNA interactions. A file in CSV (Comma-separated values) format is generated containing all the predicted miRNA/mRNA interactions found. In addition, filtered files are generated, reporting miRNA targets with different seed regions and, whenever possible, comparing the results between miRanda, RNAHybrid and PITA.

miRP also presents scripts designed to compare sequences with variation sites, seeking for the best miRNA/mRNA interaction for each miRNA in a specific mRNA polymorphic region, in order to detect the influence of variation sites in the miRNA/mRNA hybridization. Additional scripts for filtering and converting files are also available.

Scripts interface and installation

The miRP package is all written in Perl, a platform-independent programming language. Although miRP is platform-independent, Operational System specific binaries of the miRanda, RNAHybrid and PITA are necessary. The scripts were developed to work with any UNIX compatible system.

Currently, miRP works with miRanda (**version 3.3a**), RNAhybrid (**version 2.1.1**) and PITA (**version 6 from 31-Aug-2008**). Please check for miRP updates in "bioinfo.icb.ufg.br". You need to download and install it properly before use MIRP.

To install miRP, you must decompress the distribution file and put the miRP folder in a place that is easily accessed by Terminal. The miRP folder structure and files are as follows. Each of the files will be discussed separately in further sections.

> mirbase (folder)
> mature_human.fas

> source (folder)
> exampleA.fas
> exampleB.fas
> miRP_1.01_docs.pdf (this file)
> mirp-csv.pl (script)
> mirp-fastafilter-file.pl (script)
> mirp-fastafilter-string.pl (script)
> mirp-hybrid.pl (script)
> mirp-miranda.pl (script)
> mirp-pita.pl (script)
> mirp-runner.pl (script)
> mirp-seqsplit.pl (script)
> mirp.pm (mirp application file)
> mirp-snp.pl (script)
> mirp-setup.pl (script)

Getting started

The first thing to do with miRP is setup your system, assuming that you already have a working copy of the miRanda, RNAHybrid and PITA. To setup your system and generate the configuration file needed to use miRP, you have to use the script "mirp-setup.pl" as follows:

> perl mirp-setup.pl

It will be asked about the path to the miRanda, RNAHybrid and PITA applications. You may type, or drag and drop the main application files (miranda, RNAhybrid and pita_prediction.pl files) in the appropriate field. Mirp-setup will check if the applications are working properly. Once it is completed, a file named "setup.txt" will be created in the miRP folder with all the parameters needed to use miRP.

The structure of the setup file is as follows. You may further edit this file (or make a copy with different names) to change the parameters. An explanation of each of these parameter values is given.

split_length = [50]
# The size of the subsequences that will be generated. Zero (0) indicates that you do not want to create subsequences and instead you will use the entire sequence as a whole.

split_evolution = [5]
# Each of the subsequences starts 5 nucleotides further than the previous one.

rnahybrid_path = [/Users/user/Apps/RNAhybrid/RNAhybrid]
# Path to the RNAHybrid binary.

hybrid_utr = [3utr_human]
# The type of mRNA that is being used. By default, the configuration is set to a human 3'UTR. Please refer to the RNAhybrid documentation to change this value if necessary, such as 3utr_worm.

hybrid_min_energy = [-10]
# The minimum MFE to be considered by RNAHybrid (energy cut-off).

hybrid_multihits = [10]
# Maximum number of hits by each RNAHybrid run for each miRNA (1 to 10).

hybrid_constraint = [0]
# Helix constraint, forcing duplexes to have a helix in specific nucleotides. Zero means that you do not want to use it. To use it, you must specify the interval, e.g., [2,7]. Please refer to the RNAHybrid documentation, option "-f".

hybrid_maxloop = [no]
# Maximum internal loop size, in which "no" means that you do not want to use it. Please refer to the RNAHybrid documentation, option "-u".

hybrid_maxbulge = [no]
# Maximum bulge loop size, in which "no" means that you do not want to use it. Please refer to the RNAHybrid documentation, option "-v".

hybrid_seed_tolerance = [3]
# Number of nucleotides to be considered when deciding if two hits present the same seed. This decision uses the last paired nucleotide in the seed region as a parameter.

miranda_path = /Users/user/Apps/miranda/miranda]
# Path to the miRanda binary.

miranda_min_energy = [-10]
# The minimum MFE to be considered by miRanda (energy cut-off).

miranda_min_score = [50]
# The minimum Score considered by miRanda (score cut-off).

miranda_gap_open = [-9]
# The gap penalty used by miRanda.

miranda_gap_extension = [-4]
# The extension penalty used by miRanda.

miranda_seed_tolerance = [3]
# Number of nucleotides to be considered when deciding if two hits present the same seed. This decision uses the last paired nucleotide in the seed region as a parameter.

comparison_seed_tolerance = [3]
# Number of nucleotides to be considered when deciding if two hits present the same seed, considering only the comparison among miRanda, RNAHybrid and PITA. This decision uses the last paired nucleotide in the seed region as a parameter (for miRanda and RNAHybrid) and the last base of the seed region (for PITA).

pita_path = [/Users/user/Apps/pita/pita_prediction.pl]
# Path to the PITA main script.

pita_seed_tolerance = [3]
# Number of nucleotides to be considered when deciding if two hits present the same seed. This decision uses the last seed nucleotide as a parameter.

Performing a systematic scan of a mRNA sequence

miRP-Runner

The mirp-runner is a script that automates miRNA target prediction and generates lists of targets in CSV format. It uses miRanda, RNAHybrid and PITA to predict the targets.

To use mirp-runner, you need a mRNA sequence in fasta format (e.g., the file presented at source/exampleA.fas), a database of miRNA sequences in fasta format (e.g. the file presented at mirbase/mature_human.fas) and a setup file such as the one created by the script mirp-setup.pl.

Attention: mirp-runner only accepts single mRNA sequences in fasta format. Fasta files containing several mRNA sequences will not be properly processed. The microRNA database may be a multi-sequence fasta format, as presented in the example file "mirbase/mature_human.fas".

To run this mirp-runner, use:

> perl mirp-runner.pl <mRNA_sequence> <miRNA_database> <setup_file>

> e.g., perl mirp-runner.pl /source/exampleA.fas /mirbase/mature_human.fas setup.txt

The script will generate an output folder, usually named "miRP-Runner-(date,hour)-mRNA_sequence_name", that contains all the output files. The structure of this folder is as follows:

> miranda (folder)
– Contains the miRanda outputs for all the generated subsequences.

> hybrid (folder)
– Contains the RNAHybrid outputs for all the generated subsequences.

> pita (folder)
– Contains the PITA outputs for all the generated subsequences.

> results (folder)
> miranda-results-full-records-sorted.csv
> miranda-results-full-records.csv
> miranda-results-seed.csv
> hybrid-results-full-records-sorted.csv
> hybrid-results-full-records.csv
> hybrid-results-seed.csv
> hybrid-results-3end.csv
> pita-results-full-records-sorted.csv
> pita-results-full-records.csv
> pita-results-seed.csv
> miRNA-compared.csv
> best-mirna.csv

> source (folder)
> a copy of the mRNA input sequence
> a fasta file (_split) with the subsequences used

> split (folder)
> Contains single fasta files with all the subsequences

> tmp (folder)
> an internal file (id.txt). Do not delete or change this file.
> a copy of the miRNA database used, names mirbase.txt
> a list of the miRNA names
> a copy of the setup file used

The split folder

The first thing that mirp-runner does is create subsequences as specified in the setup file. As default, mirp-runner creates several overlapping subsequences of 50 nucleotides each. Each of these subsequences starts 5 nucleotides further than the previous one. This step maximizes the ability of the algorithms to predict all possible miRNA/mRNA interactions. Each of the subsequences generated is kept in the "split" folder and will be used by miRanda, RNAHybrid and PITA.

The results folder

The files in the results folder are CSV format files, using "," to separate fields. You may use any spreadsheet software to edit, filter or visualize these files. Each of these files will be presented separately.

- miranda-results-full-records-sorted.csv

This file contains all the miRNA/mRNA targets predicted by miRanda, considering all the subsequences. The CSV file has no header, but the fields are as presented bellow (using the given exampleA.fas and mature_human.fas). It starts with the miRNA name, followed by the sequence name and segment used in the scan, the alignment start position, the alignment end position, the position of the last paired nucleotide in the seed region (considering as seed nucleotides 2 to 7), the MFE value and the Score.

miRNA_name	sequence_and_segment	start position	end position	last paired base	MFE	Score
hsa-let-7a-2-3p	exampleA_segm_000001	20	41	40	-10.86	108
hsa-let-7a-2-3p	exampleA_segm_000002	20	41	40	-10.86	108
hsa-let-7a-2-3p	exampleA_segm_000003	20	41	40	-10.86	108
hsa-let-7a-2-3p	exampleA_segm_000004	20	41	40	-10.86	108
hsa-let-7a-2-3p	exampleA_segm_000005	21	41	40	-10.86	108
hsa-let-7a-2-3p	exampleA_segm_000006	26	41	40	-10.86	108
hsa-let-7a-2-3p	exampleA_segm_000007	31	41	40	-10.25	108

- miranda-results-seed.csv

This file is a filter of the “miranda-results-full-records-sorted.csv” presented above. For this file, only miRNA/mRNA targets with different seeds were kept (considering the last paired base of the seed region). In cases of different hits with similar or close position for the last paired base of the seed region (considering the tolerance given in the setup file), only the hit with the lower MFE value is parsed to the “miranda-results-seed.csv” file. The structure of this file is the same as the one presented above for the “miranda-results-full-records-sorted” file.

- hybrid-results-full-records-sorted.csv

This file contains all the miRNA/mRNA targets predicted by RNAHybrid, considering all the subsequences. The CSV file has no header, but the fields are presented bellow (using the given exampleA.fas and mature_human.fas). It starts with the miRNA name, followed by the sequence name and segment used in the scan, the alignment start position, the alignment end position, the position of the last paired nucleotide in the seed region (considering as seed nucleotides 2 to 7), the MFE value, the absolute number of paired seed nucleotides, the percentage of pairing between miRNA and the mRNA and probability.

In some cases, the “last paired base” value is 0. This indicates that this specific hit does not present any paired base in the seed region.

miRNA_name	sequence_and_segment	start position	end position	last paired base	MFE	paired seed nucleotides	% pairing	probability
hsa-let-7a-2-3p	exampleA_segm_000001	33	42	41	-14.4	6	0.364	0.999
hsa-let-7a-2-3p	exampleA_segm_000002	33	42	41	-14.4	6	0.364	0.999
hsa-let-7a-2-3p	exampleA_segm_000003	33	42	41	-14.4	6	0.364	0.999
hsa-let-7a-2-3p	exampleA_segm_000004	33	42	41	-14.4	6	0.364	0.999
hsa-let-7a-2-3p	exampleA_segm_000005	33	42	41	-14.4	6	0.364	0.999
hsa-let-7a-2-3p	exampleA_segm_000006	33	42	41	-14.4	6	0.364	0.999
hsa-let-7a-2-3p	exampleA_segm_000007	33	42	41	-14.4	6	0.364	0.999
hsa-let-7a-2-3p	exampleA_segm_000003	40	60	60	-10.6	4	0.5	1
hsa-let-7a-2-3p	exampleA_segm_000004	40	62	61	-13.7	5	0.545	1
hsa-let-7a-2-3p	exampleA_segm_000005	40	62	61	-13.7	5	0.545	1
hsa-let-7a-2-3p	exampleA_segm_000006	40	62	61	-13.7	5	0.545	1
hsa-let-7a-2-3p	exampleA_segm_000007	40	62	61	-13.7	5	0.545	1
hsa-let-7a-2-3p	exampleA_segm_000008	40	62	61	-13.9	5	0.545	1
hsa-let-7a-2-3p	exampleA_segm_000009	41	62	61	-13.7	5	0.545	1
hsa-let-7a-2-3p	exampleA_segm_000010	46	62	61	-13.5	5	0.409	1
hsa-let-7a-2-3p	exampleA_segm_000009	83	88	87	-10	2	0.182	1
hsa-let-7a-2-3p	exampleA_segm_000010	83	95	94	-11.5	6	0.364	1

- hybrid-results-seed.csv

This file is a filter of the "hybrid-results-full-records-sorted.csv". For this file, only miRNA/mRNA targets with different seeds were kept (considering the last paired base of the seed region). In cases of different hits with similar or close position for the last paired base of the seed region (considering the tolerance given in the setup file), only the hit with the lower MFE value is considered in the "hybrid-results-seed.csv" file. The structure of this file is the same as the one presented above for the "hybrid-results-full-records-sorted" file.

- hybrid-results-3end.csv

- This file is a filter of the "hybrid-results-full-records-sorted.csv". For this file, only miRNA/mRNA targets with no paired nucleotide in the seed region is considered. In cases of different hits with similar or close position for the start position of the alignment (considering the tolerance given in the setup file), only the hit with the lower MFE value is parsed to the "hybrid-results-3end.csv" file. The structure of this file is the same presented above for the "hybrid-results-full-records-sorted" file. Since miRanda does not consider targets with no paired seed, this file is not available for miRanda.

- pita-results-full-records-sorted.csv

This file contains all the miRNA/mRNA targets predicted by PITA, considering all the subsequences. The CSV file has no header, but the fields are presented bellow (using the given exampleA.fas and mature_human.fas). It starts with the miRNA name, followed by the sequence name and segment used in the scan, the seed end alignment position, the seed start alignment position and the ddG value.

miRNA_name	sequence_and_segment	seed end position	seed start position	ddG
hsa-let-7e-5p	exampleA_segm_000001	30	22	-7.89
hsa-let-7g-3p	exampleA_segm_000008	63	55	-8.28
hsa-let-7i-3p	exampleA_segm_000003	41	34	-4.17
hsa-miR-1178-5p	exampleA_segm_000001	31	23	-7.37
hsa-miR-1178-5p	exampleA_segm_000006	75	68	-14.33
hsa-miR-1182	exampleA_segm_000001	25	17	-12.1
hsa-miR-1182	exampleA_segm_000006	75	67	-18.13
hsa-miR-1224-5p	exampleA_segm_000009	83	77	-16.03
hsa-miR-1225-5p	exampleA_segm_000009	65	58	-20.72
hsa-miR-1228-5p	exampleA_segm_000002	53	45	-11.51
hsa-miR-1229-5p	exampleA_segm_000002	53	45	-15.31
hsa-miR-1234-3p	exampleA_segm_000009	88	80	-2.72
hsa-miR-1234-5p	exampleA_segm_000003	58	50	-17.38

- pita-results-seed.csv

This file is a filter of the "pita-results-full-records-sorted.csv". For this file, only miRNA/mRNA targets with different seeds were kept (considering the last seed nucleotide). In cases of different hits with similar or seed close position (considering the tolerance given in the setup file), only the hit with the lower ddG value is considered in the "pita-results-seed.csv" file. The structure of this file is the same as the one presented above for the "pita-results-full-records-sorted" file.

- miRNA-compared.csv

- This file is a comparison between miRanda, RNAHybrid and PITA results. On the right side of the CSV file is the data from miRanda, in the middle is the data of RNAhybrid and in the left side is the data from PITA. Data is presented according to the seed position, considering the aligned position of the last seed nucleotide. If all methods inferred the same miRNA target and these hits present the same or close seed position (considering the tolerance value in the setup file – key "comparison_seed_tolerance"), the miRanda, RNAHybrid and PITA data are presented together. If a miRNA target was not predicted a method, this data is filled with zeros. This file indicates the compatibility between miRanda, RNAHybrid and PIta. The CSV file has no header, but the fields are presented bellow (using the given exampleA.fas and mature_human.fas).

RNAHybrid data					miRanda data					Pita data			
miRNA	alignment start position	alignment end position	last paired base	MFE	miRNA	alignment start position	alignment end position	last paired base	MFE	miRNA	seed start position	seed end position	ddG
hsa-let-7e-5p	26	32	31	-12	hsa-let-7e-5p	10	30	29	-11.63	hsa-let-7e-5p	22	30	-7.89
hsa-let-7e-5p	9	33	32	-18.9	hsa-let-7e-5p	16	37	35	-11.24	hsa-let-7e-5p	0	0	0
hsa-let-7e-5p	33	50	50	-16.6	hsa-let-7e-5p	32	50	47	-11.4	hsa-let-7e-5p	0	0	0
hsa-let-7e-5p	33	55	54	-19.4	hsa-let-7e-5p	32	55	52	-14.32	hsa-let-7e-5p	0	0	0
hsa-let-7e-5p	33	65	64	-19.9	hsa-let-7e-5p	48	69	66	-10.79	hsa-let-7e-5p	0	0	0
hsa-let-7e-5p	63	75	75	-10.9	hsa-let-7e-5p	55	75	74	-13.25	hsa-let-7e-5p	0	0	0
hsa-let-7e-5p	66	80	80	-16.7	hsa-let-7e-5p	61	82	81	-18.99	hsa-let-7e-5p	0	0	0
hsa-let-7e-5p	55	83	82	-23.6	hsa-let-7e-5p	0	0	0	0	hsa-let-7e-5p	0	0	0

- best-mirna.csv

This file is a filter of the "miRNA-compared.csv" in which only miRNA targets that were predicted by the three algorithms are listed. The structure of this file is the same as the one presented above for the "miRNA-compared.csv" file.

Using mirp-runner without a prediction method

The mirp-runner script automates miRNA target prediction by using three different algoritms. However, if one algorithm is not properly installed and not available in the setup.txt file, mirp-runner will skip the prediction step using this algorithm as well as skip the comparative analyses, not generating files best-mirna.csv and miRNA-compared.csv.

miRP-miRanda, miRP-Hybrid and miRP-Pita

The mirp-miRanda is a script that automates miRNA target prediction and generated list of targets in CSV format. It uses only miRanda to predict the targets.

The mirp-Hybrid is a script that automates miRNA target prediction and generates a list of targets in CSV format. It uses only RNAHybrid to predict the targets.

The mirp-Pita is a script that automates miRNA target prediction and generates a list of targets in CSV format. It uses only Pita to predict the targets.

These scripts are small versions of miRP-Runner script, because they use only one algorithm to predict the targets. Besides that, they perform the same analyses as described for miRP-Runner, but no comparison files are made. The folder outputs present the same structure as miRP-Runner, but the results for only one method is presented.

Looking for miRNA that are influenced by SNPs or haplotypes

miRP-SNP

Mirp-snp is a script that compares two *mirp-runner*, *mirp-miranda, mirp-pita* or *mirp-hybrid* runs that used different sequences of the same size but with few nucleotide differences. In other words, it is a script to look for miRNAs that might be influenced by the presence of SNPs or specific haplotypes.

Attention: mirp-snp is only suitable to compare sequences with the same size. Deletions and insertions will be accepted, but the results will be messed up.

To use mirp-snp, first you need to run mirp-runner, mirp-miranda or mirp-hybrid for the two sequences you want to compare. Then, you have to run mirp-snp as following:

> perl mirp-snp.pl

The script will ask you for the first folder you want to use in the comparison. Just type or drag and drop the folder you want in the appropriate field. Then, mirp-runner will ask you about the second folder you want to use in the comparison. Again, just type or drag and drop the folder you want in the appropriate field.

It is mandatory that the folders you want to compare came from the same script, eg., mirp-runner against mirp-runner, mirp-miranda against mirp-miranda, etc…

After you inform both the folders for comparison, the script will ask about the SNP position or a position interval you want to use. In our example, you must input number 20, indicating that the SNP is at position 20. It will filter out all the miRNA targets in which their miRNA/mRNA alignment (for miRanda and RNAHybrid) or the seed position (for Pita) includes this position (this position must be the first in the alignment, the last or in between). In order words, it will select miRNA that target the SNP region.

If there is more than one SNP in both sequences you are comparing, you may use two approaches:

a) Specify an interval despite of a specific position, such as (20-25). In this case, the script will select any miRNA target that passes by this interval.
b) Run mirp-snp as many times as you want specifying different SNP positions. However, if the miRNA are close enough that haplotypes might influence the miRNA target, you should use option a.

As soon as the position(s) to scan for miRNAs is(are) set, the script will ask you about the seed position tolerance. This value is used to decide if two hits present the same seed region, keeping then only the hit with the lower MFE or ddG. It is recommended that you use the same value set for the "comparison-seed-tolerance" key in the setup file.

Once the script finishes all computations, you will find an output folder, usually named "miRP-SNP-(date,hour)", that contains all the files and databases created. The structure of this folder is as follows:

> results (folder)
– Contains all the CSV files created (see below description of each file).

> source (folder)
– Contains two folders, *data1* and *data2*, in which a copy of the files from the folders that are being compared are kept.

> tmp (folder)
> An internal file (id.txt). Do not delete or change this file.

The results folder

The files in the results folder are CSV files, using "," to separate fields. You may use any spreadsheet software to edit, filter or visualize these files. Each of these files will be presented separately.

- miranda-data1-SNP.csv and miranda-data2-SNP.csv (when comparing mirp-runner or mirp-miranda runs)

These files contain all the miRNA/mRNA targets predicted by miRanda that pass by the position you specified, in which *data1* provides the targets found in the first folder you informed (or the first sequence) and *data2* provide the same data for the second folder (or the second sequence). However, since the script seeks for most stable configuration of a miRNA that passes by the position you informed, only single hits are present, i.e., for each miRNA that may target the SNP region, only the most stable hit is presented.

- hybrid-data1-SNP.csv and hybrid-data2-SNP.csv (when comparing mirp-runner or mirp-hybrid runs)

These files contain all the miRNA/mRNA targets predicted by RNAHybrid that pass by the position you specified, in which *data1* provides the targets found in the first folder you informed (or the first sequence) and *data2* provide the same data for the second folder (or the second sequence). However, since the script seeks for most stable configuration of a miRNA that passes by the position you informed, only single hits are present, i.e., for each miRNA that may target the SNP region, only the most stable hit is presented.

- pita-data1-SNP.csv and pita-data2-SNP.csv (when comparing mirp-runner or mirp-pita runs)

These files contain all the miRNA/mRNA targets predicted by PITA that pass by the position you specified, in which *data1* provides the targets found in the first folder you informed (or the first sequence) and *data2* provide the same data for the second folder (or the second sequence). The structure of this file is the same one described for the "pita-results-full-records-sorted.csv" file. However, since the script seeks for most stable configuration of a miRNA that passes by the position you informed, only single hits are present, i.e., for each miRNA that may target the SNP region, only the most stable hit is presented.

- miranda-compared-SNP.csv (when comparing mirp-runner or mirp-miranda runs)

This file is a comparison between the miRNA targets predicted by miRanda between data1 (the first folder) and data2 (the second folder). To pair the predicted miRNA/mRNA interactions between both runs, the script uses the last paired nucleotide in the seed region as a parameter, always considering the value informed as a tolerance value (the last paired base in the seed region must be similar or close enough). If the same miRNA target was inferred for both sequences (data1 and data2), these hits are presented together. If a miRNA target was predicted for only one sequence, only the data for this sequence is presented. In addition, the absolute difference between the MFE value for data1 and data2 is calculated and presented. This difference may indicate how much this variation site does influence miRNA binding. When a miRNA target is inferred for only one sequence, the MFE difference remains ZERO since the value would not be the correct one. The CSV file containing this comparison has no header, but the fields are presented bellow (using the given exampleA.fas, example.fas and mature_human.fas as the miRNA database).

DATA1 - FIRST FOLDER - EXAMPLE-A							DATA2 - SECOND FOLDER - EXAMPLE-B								
**miRNA name**	**sequence_and_segment**	**start position**	**end position**	**last paired base**	**MFE**	**Score**	**miRNA name**	**sequence_and_segment**	**start position**	**end position**	**last paired base**	**MFE**	**Score**	**MFE difference**	**Note**
hsa-let-7a-2-3p	exampleA_segm_000001	20	41	40	-10.86	108	hsa-let-7a-2-3p	exampleB_segm_000001	20	41	40	-10.86	108	0	mirp-snp
hsa-let-7a-5p	exampleA_segm_000001	16	37	35	-12.8	63	hsa-let-7a-5p	exampleB_segm_000001	16	37	35	-12.53	63	0.27	mirp-snp
hsa-let-7c	exampleA_segm_000001	16	37	35	-12.8	63	hsa-let-7c	exampleB_segm_000001	16	37	35	-12.53	63	0.27	mirp-snp
hsa-let-7d-5p	exampleA_segm_000001	16	37	35	-13.49	63	hsa-let-7d-5p	exampleB_segm_000001	16	37	35	-13.37	63	0.12	mirp-snp
hsa-let-7e-5p	exampleA_segm_000001	10	30	29	-11.63	102	hsa-let-7e-5p	exampleB_segm_000001	10	30	29	-11.65	102	0.02	mirp-snp
hsa-let-7i-3p	exampleA_segm_000001	20	41	40	-12.73	125	hsa-let-7i-3p	exampleB_segm_000001	20	41	40	-12.73	125	0	mirp-snp
hsa-miR-103a-3p	exampleA_segm_000001	5	27	25	-12.05	70	hsa-miR-103a-3p	exampleB_segm_000001	5	27	25	-15.57	60	3.52	mirp-snp
hsa-miR-106b-3p	exampleA_segm_000001	20	41	39	-10.28	86	hsa-miR-106b-3p	exampleB_segm_000001	2	23	21	-10.96	68	0.68	mirp-snp
hsa-miR-107	exampleA_segm_000001	5	27	25	-12.05	70	hsa-miR-107	exampleB_segm_000001	5	27	25	-14.67	60	2.62	mirp-snp
hsa-miR-1178-5p	exampleA_segm_000001	14	31	29	-12.81	109	hsa-miR-1178-5p	exampleB_segm_000001	14	31	29	-13.71	109	0.9	mirp-snp
hsa-miR-1180	exampleA_segm_000001	1	21	20	-11.24	60	hsa-miR-1180	exampleB_segm_000001	6	27	22	-10.78	72	0.46	mirp-snp
hsa-miR-1182	exampleA_segm_000001	9	32	31	-17.29	84	hsa-miR-1182	exampleB_segm_000001	9	32	31	-18.34	84	1.05	mirp-snp
hsa-miR-1183	exampleA_segm_000001	15	43	41	-17.14	88	hsa-miR-1183	exampleB_segm_000001	19	43	41	-19.37	86	2.23	mirp-snp
hsa-miR-1184	exampleA_segm_000001	20	42	41	-10.64	100	hsa-miR-1184	exampleB_segm_000001	20	42	41	-10.64	100	0	mirp-snp
hsa-miR-1185-1-3p	exampleA_segm_000001	9	30	25	-10.62	70	hsa-miR-1185-1-3p	exampleB_segm_000001	9	30	25	-11.79	70	1.17	mirp-snp
hsa-miR-1185-2-3p	exampleA_segm_000001	9	30	25	-10.62	70	hsa-miR-1185-2-3p	exampleB_segm_000001	9	30	25	-10.85	70	0.23	mirp-snp
hsa-miR-1202	exampleA_segm_000001	19	41	40	-18.33	74	hsa-miR-1202	exampleB_segm_000001	19	41	40	-18.33	74	0	mirp-snp
hsa-miR-1203	exampleA_segm_000001	13	33	30	-11.74	92	hsa-miR-1203	exampleB_segm_000001	13	33	30	-15.34	88	3.6	mirp-snp
hsa-miR-1205	exampleA_segm_000001	12	29	25	-15.29	97	hsa-miR-1205	exampleB_segm_000001	12	29	25	-15.02	93	0.27	mirp-snp
hsa-miR-1207-5p	exampleA_segm_000001	9	28	27	-16.27	116	hsa-miR-1207-5p	exampleB_segm_000001	9	28	27	-17.32	116	1.05	mirp-snp
hsa-miR-122-5p	exampleA_segm_000001	10	31	30	-11.88	100	hsa-miR-122-5p	exampleB_segm_000001	10	31	30	-11.09	100	0.79	mirp-snp
hsa-miR-1224-5p	exampleA_segm_000001	16	34	32	-16.12	109	hsa-miR-1224-5p	exampleB_segm_000002	9	27	26	-19.56	101	3.44	mirp-snp

- *hybrid-compared-SNP.csv (when comparing mirp-runner or mirp-hybrid runs)*

This file is a comparison between the miRNA targets predicted by RNAHybrid between data1 (the first folder) and data2 (the second folder). To pair the predicted miRNA/mRNA interactions between both runs, the script uses the last paired nucleotide in the seed region as a parameter, always considering the value informed as a tolerance value (the last paired base in the seed region must be similar or close enough). If a same miRNA target was inferred for both sequences (data1 and data2), these hits are presented together. If a miRNA target was predicted for only one sequence, only the data for this sequence is presented. In addition, the absolute difference between the MFE value for data1 and data2 is calculated and presented. When a miRNA target is inferred for only one sequence, the MFE difference remains ZERO since the value would not be the correct one. The CSV file containing this comparison has no header, but the fields are presented bellow (using the given exampleA.fas, example.fas and mature_human.fas as the miRNA database).

DATA1 - FIRST FOLDER - EXAMPLE-A									DATA2 - SECOND FOLDER - EXAMPLE-B										
**miRNA name**	**sequence_and_segment**	**start position**	**end position**	**last paired base**	**MFE**	**Paired seed nucleotides**	**% pairing**	**probability**	**miRNA name**	**sequence_and_segment**	**start position**	**end position**	**last paired base**	**MFE**	**Paired seed nucleotides**	**% pairing**	**probability**	**MFE Difference**	**Note**
hsa-let-7a-5p	exampleA_segm_000001	9	33	32	-19.8	5	0.727	0.117	hsa-let-7a-5p	exampleB_segm_000001	9	33	32	-19.8	5	0.727	0.117	0	mirp-snp
hsa-let-7b-5p	exampleA_segm_000001	9	33	32	-20.9	5	0.773	0.076	hsa-let-7b-5p	exampleB_segm_000001	9	33	32	-20.9	5	0.773	0.076	0	mirp-snp
hsa-let-7c	exampleA_segm_000001	9	33	32	-19.8	5	0.727	0.135	hsa-let-7c	exampleB_segm_000001	9	33	32	-19.8	5	0.727	0.135	0	mirp-snp
hsa-let-7d-5p	exampleA_segm_000001	9	34	33	-19.8	5	0.727	0.132	hsa-let-7d-5p	exampleB_segm_000001	9	34	33	-19.8	5	0.727	0.132	0	mirp-snp
hsa-let-7e-5p	exampleA_segm_000001	9	33	32	-18.9	4	0.682	0.24	hsa-let-7e-5p	exampleB_segm_000001	9	33	32	-18.9	4	0.682	0.24	0	mirp-snp
hsa-let-7f-2-3p	exampleA_segm_000001	15	29	28	-11.5	1	0.364	1	hsa-let-7f-2-3p	exampleB_segm_000001	15	29	28	-10.4	1	0.364	1	1.1	mirp-snp
hsa-let-7f-5p	exampleA_segm_000001	9	33	32	-15.9	5	0.727	0.822	hsa-let-7f-5p	exampleB_segm_000001	9	33	32	-16	5	0.682	0.8	0.1	mirp-snp
hsa-let-7g-3p	exampleA_segm_000001	14	42	41	-14.8	6	0.714	0.993	hsa-let-7g-3p	exampleB_segm_000001	14	42	41	-18.9	6	0.81	0.268	4.1	mirp-snp
hsa-let-7g-5p	exampleA_segm_000001	5	33	32	-17.4	5	0.727	0.479	hsa-let-7g-5p	exampleB_segm_000001	5	33	32	-17.4	5	0.727	0.479	0	mirp-snp
hsa-let-7i-5p	exampleA_segm_000001	9	33	32	-17.6	5	0.727	0.462	hsa-let-7i-5p	exampleB_segm_000001	9	33	32	-17.6	5	0.727	0.462	0	mirp-snp
hsa-miR-101-5p	exampleA_segm_000001	5	29	28	-12.1	6	0.5	1	hsa-miR-101-5p	exampleB_segm_000001	14	26	0	-13.2	0	0.364	1	1.1	mirp-snp
hsa-miR-103a-3p	exampleA_segm_000001	13	40	39	-18.1	3	0.609	0.475	hsa-miR-103a-3p	exampleB_segm_000001	13	40	39	-24.3	3	0.696	0.01	6.2	mirp-snp
hsa-miR-105-3p	exampleA_segm_000001	7	24	23	-11.7	2	0.455	1	hsa-miR-105-3p	exampleB_segm_000002	18	51	50	-15.7	3	0.636	0.922	4	mirp-snp
hsa-miR-106a-3p	exampleA_segm_000001	15	42	41	-14.6	4	0.682	0.987	hsa-miR-106a-3p	exampleB_segm_000001	15	42	41	-14.3	4	0.682	0.996	0.3	mirp-snp
hsa-miR-106b-3p	exampleA_segm_000001	14	40	39	-21.3	5	0.591	0.082	hsa-miR-106b-3p	exampleB_segm_000001	14	40	39	-21.6	5	0.591	0.068	0.3	mirp-snp
hsa-miR-107	exampleA_segm_000001	17	40	39	-17.9	3	0.565	0.521	hsa-miR-107	exampleB_segm_000001	18	40	39	-23.2	3	0.565	0.021	5.3	mirp-snp
hsa-miR-10a-3p	exampleA_segm_000001	20	26	0	-12.4	0	0.227	1	hsa-miR-10a-3p	exampleB_segm_000001	20	26	0	-12.1	0	0.227	1	0.3	mirp-snp

- *pita-compared-SNP.csv (when comparing mirp-runner or mirp-pita runs)*

This file is a comparison between the miRNA targets predicted by PITA between data1 (the first folder) and data2 (the second folder). To pair the predicted miRNA/mRNA interactions between both runs, the script uses the seed end position as a parameter, always considering the value informed as a tolerance value. If a same miRNA target was inferred for both sequences (data1 and data2), these hits are presented together. If a miRNA target was predicted for only one sequence, only the data for this sequence is presented. In addition, the absolute difference between the ddG value for data1 and data2 is calculated and presented. When a miRNA target is inferred for only one sequence, the ddG difference remains ZERO since the value would not be the correct one. The CSV file containing this comparison has no header, but the fields are presented bellow (using the given exampleA.fas, example.fas and mature_human.fas as the miRNA database).

DATA1 - FIRST FOLDER - EXAMPLE-A					DATA2 - SECOND FOLDER - EXAMPLE-B						
**miRNA**	**sequence and segment**	**end seed potision**	**start seed position**	**ddG**	**miRNA**	**sequence and segment**	**end seed potision**	**start seed position**	**ddG**	**ddG difference**	**Note**
hsa-miR-182-3p	exampleA_segm_000001	23	15	-7.39	hsa-miR-182-3p	exampleB_segm_000001	23	15	-5.24	2.15	mirp-snp
hsa-miR-193b-5p	exampleA_segm_000001	24	16	-12.93	hsa-miR-193b-5p	exampleB_segm_000001	24	16	-12.88	0.05	mirp-snp
hsa-miR-218-2-3p	exampleA_segm_000001	25	17	-9.6	hsa-miR-218-2-3p	exampleB_segm_000001	25	17	-7.45	2.15	mirp-snp
hsa-miR-23a-5p	exampleA_segm_000001	24	16	-18.93	hsa-miR-23a-5p	exampleB_segm_000001	24	16	-16.78	2.15	mirp-snp
hsa-miR-23b-5p	exampleA_segm_000001	24	16	-10.23	hsa-miR-23b-5p	exampleB_segm_000001	24	16	-8.08	2.15	mirp-snp
hsa-miR-3127-5p	exampleA_segm_000001	27	19	-13.67	hsa-miR-3127-5p	exampleB_segm_000001	27	19	-18.13	4.46	mirp-snp
hsa-miR-542-5p	exampleA_segm_000001	26	18	-16.2	hsa-miR-542-5p	exampleB_segm_000001	26	18	-14.05	2.15	mirp-snp
0	0	0	0	0	hsa-miR-1245b-5p	exampleB_segm_000001	25	17	-5.45	0	mirp-snp
0	0	0	0	0	hsa-miR-1266	exampleB_segm_000001	28	20	-14.62	0	mirp-snp
0	0	0	0	0	hsa-miR-1296	exampleB_segm_000001	26	18	-6.75	0	mirp-snp
0	0	0	0	0	hsa-miR-135a-5p	exampleB_segm_000001	25	17	-2.95	0	mirp-snp

Other supporting scripts

A series of other MIRP scripts is available to assist miRNA target prediction by using miRanda, RNAHybrid and PITA. The scripts, their usage and description are given bellow.

mirp-csv

This is a script that converts any miRanda, RNAHybrid or PITA output to a CSV file in the same format presented above. To use it, type “perl mirp-csv.pl [the_input_file] [the_output_file]. The input file is any output of the miRanda, RNAHybrid or PITA. The script will automatically set itself to the type of output you are pointing to. The output file is the name of the CSV file you want to create.

mirp-fastafilter-file.pl

This is a script that filters out from a multi-sequence FASTA file only the sequences that are listed in another text file. To use it, type “perl mirp-fastafilter-file.pl <input.fas> <list file> <output file>”. The input file is any multi-sequence FASTA file, the output file is the name of the list of sequences you want to get from the original fasta file and the ouput file is the name of the new fasta file you want to create.

mirp-fastafilter-string.pl

This is a script that filters out from a multi-sequence FASTA file only the sequences whose name match with the string provided. To use it, type “perl mirp-fastafilter-string.pl <input.fas> <string to search> <output file>”.

mirp-seqsplit.pl

This is a script that split a given fasta format sequence into several small sequences as it is done by mirp-runner, mirp-hybrid, mirp-pita and mirp-miranda. To use it, type “perl mirp-seqsplit.pl <input.fas> <size_of_each_subsequence> <number_of_nucleotides_increased_in_each_subsequence>”.

Known issues and bug reporting

Currently the MIRP package is working only with UNIX-compatible systems. This is caused mainly by the lack of a Windows© binary of the miRanda and PITA. However, by editing the setup file manually, you may use mirp-hybrid and mirp-snp under a Windows© environment.

Fasta files that were edited under a Windows© environment may not work properly. In this case, open this sequence in any text-edit software under a UNIX compatible system and save the file with a different name.

Please report any bug or suggestions to the author at the MIRP website.

Version history

Version 1.0: The first release of MIRP.

Version 1.01: I bug in which RNAhybrid results were not properly filtered was fixed.

References

(1) John, B., A. J. Enright, et al. (2004). "Human MicroRNA targets." PLoS Biol **2**(11): 1862-1879.

(2) Kruger, J. and M. Rehmsmeier (2006). "RNAhybrid: microRNA target prediction easy, fast and flexible." Nucleic Acids Res **34**(Web Server issue): W451-454.

(3) Kertesz, M., N. Iovino, et al. (2007). "The role of site accessibility in microRNA target recognition." Nat Genet **39**(10): 1278-1284.
