# Supplementary material for: 3′ UTR G-quadruplexes regulate miRNA binding
Source: RNA. 2017 Aug;23(8):1172–9. doi: 10.1261/rna.060962.117 (PMC5513062; doi:10.1261/rna.060962.117)
Supplement: Supplemental Material [file supp_060962.117_Supplemental_Material.pdf]

List of Supplementary Tables

Supplementary Table 1. **G-Quadruplexes in the 3’UTR overlapping with microRNA binding sites**

Supplementary Table 2. **Sequences of the primers used.**

List of Supplementary figures

Supplementary Figure 1. **In-line probing of ELK1, HDGF, THRB, and WNT3A.**

Supplementary Figure 2. **N-methyl Mesoporphyrin IX fluorescence assay of ELK1, HDGF, THRB, and WNT3A.**

Supplementary Figure 3. **Other tested G4 do not regulate miRNA binding.**

Supplementary Figure 4. **Effect of a mir331 inhibitor on FADS2 reporter expression.**

List of Supplementary files

Supplementary File 1. **G4 in the 3’UTR of human mRNAs**

This file contains the list of G4 found in the 3’UTR of human genes, along with information on the gene, position within the 3’UTR(1 being the first nucleotide after the stop codon), length, sequence of the G4, and the cG/cC score.

Supplementary File 2. G4 overlapping with miRNA binding sites

This file contains the list of G4 predicted to be bound by miRNAs. The alignment was made with the predicted G4 and 10 flanking nucleotides on each side, therefore the alignment position 1 refers to 10 less than the ‘’Position’’ column in Supplementary File 1.

Supplementary File 3. **Gene Ontology analysis of the predicted miRNA bound G4**

This file contains the list of enriched biological process in the G4 predicted to be overlapping miRNA binding sites.

Supplementary File 4. **Instructions for the miRP scripts**

This is the user manual for the mIRP scripts used to predict the miRNA binding sites.

Supplementary Table 1. **G-Quadruplexes in the 3'UTR overlapping with microRNA binding sites**

Genes containing at least 1 G4	Total number of G4	Genes containing at least 1 G4 predicted to overlap with 1 miRNA binding site	Total number of G4 predicted to overlap with at least 1 miRNA binding site	miRNAs predicted to bind at least 1 G4	G4/miRNA pair	miRNA binding sites predicted to bind exclusively in G4's loop
1,112	2,282	611	1,233	521	44,294	13,361

Supplementary Table 2. **Sequences of the primers used.**

Name	Sequence
T7 2G	taatacgactcactatagg
FADS2 WT	TCATGAGGTACCCCCTCCCCAGCCCCTCAGGGCAGCCTCCTCCTGAGACTCCCTCCCGCTCTGCCTCCCTGGCCtatagtgagtcgtatta
FADS2 G/A mutant	TCATGAGGTACCTTCTCTTCAGCCCCTCAGGGCAGCCTCCTCCTGAGACTCTCTCTCGCTCTGCCTCCCTGGCCtatagtgagtcgtatta
FADS2 Target mutant	TCATGAGGTACCCCCTCCCCACGTTCTCAGGGCAGCCTCCTCCTGAGACTCCCTCCCGCTCTGCCTCCCTGGCCtatagtgagtcgtatta
ELK1 WT	TATGTCCCCCATTCCTCCCTCCCAACTCCAGGGACATTGAAAGGGTCCTTTGTACCCGCCCGCAGGAAATTGGGGCCtatagtgagtcgtatta
ELK1 G/A mutant	TATGTCCCCCATTCCTCTCTCTCAACTCCAGGGACATTGAAAGGGTCCTTTGTACTCGCTCGCAGGAAATTGGGGCCtatagtgagtcgtatta
ElK1 Target mutant	TATGTCCCCCATTCCTCCCTCCCAACTGCGTTGACATTGAAAGGGTCCTTTGTACCCGCCCGCAGGAAATTGGGGCCtatagtgagtcgtatta
HDGF WT	CGCCCTCCTGTGCTGCCCCCACCCCGCATTTGAGGATTGCCCCTCCCAGGCCCCACAAGCCCACCCTGCTGTTGATGCTCCtatagtgagtcgtatta
HDGF G/A mutant	CGCCCTCCTGTGCTGCTTTCACTTCGCATTTGAGGATTGCCCCTCCCAGGCCCCACAAGCTCACTCTGCTGTTGATGCTCCtatagtgagtcgtatta
HDGF Target mutant	CGCCCTCCTGTGCTGCCCCCACCCCGCATTTGAGGTGTACCCCTCCCAGGCCCCACAAGCCCACCCTGCTGTTGATGCTCCtatagtgagtcgtatta
THRB WT	GACTACTTCCCTTTTCCCTCCCAAATAATCCCTCCCAACACAAAGAAACCtatagtgagtcgtatta
THRB G/A mutant	GACTACTTCCCTTTTCCCTCCCAAATAATCTCTCTCAACACAAAGAAACCtatagtgagtcgtatta
THRB Target mutant	GACTACTCTCTCTCTCCCTCCCAAATAATCCCTCCCAACACAAAGAAACCtatagtgagtcgtatta

Wnt3A WT	TTCCCAGGGAAGAGTCCCACCCGCGGAGAGAAGCCCCGCCCCATTGGAGCCTGGTCCCtatagtgagtcgtatta
Wnt3A G/A mutant	TTCCCAGGGAAGAGTCCCACCCGCGGAGAGAAGCTTCGCTTCATTGGAGCCTGGTCCCtatagtgagtcgtatta
Wnt3A Target mutant	TTCCCAGGGACGTTACCCACCCGCGGAGAGAAGCCCCGCCCCATTGGAGCCTGGTCCCtatagtgagtcgtatta
Mir331	GCCCCUGGGCCUAUCCUAGAA
Control miRNA	UUGUACUACACAAAAGUACUG
hsa-miR-331-3p.SpFwd_RT	GTCGTATCCAGTGCAGGGTCCGAGGTATTCGCACTGGATACGACTTCTAG
hsa-miR-16-5p.SpFwd_RT	GTCGTATCCAGTGCAGGGTCCGAGGTATTCGCACTGGATACGACCGCCAA
hsa-miR-142-3p.SpFwd_RT	GTCGTATCCAGTGCAGGGTCCGAGGTATTCGCACTGGATACGACTCCATA
hsa-let-7a-5p.SpFwd_RT	GTCGTATCCAGTGCAGGGTCCGAGGTATTCGCACTGGATACGACAACTAT
hsa-miR-331-3p.SpFwd_qpcr	GCGCCCCTGGGCCTATC
hsa-miR-16-5p.SpFwd_qpcr	GCTGTCGTAGCAGCACGTAAATA
hsa-miR-142-3p.SpFwd_qpcr	GCTGTCGTGTAGTGTTTCCTACTT
hsa-let-7a-5p.SpFwd_qpcr	GCTGTCGTGAGGTAGTAGGTTGT
shared-q-pcr_mir_rev	GTGCAGGGTCCGAGGT
Mir331 inhibitor	mU/ZEN/mUmCmUmAmGmGmAmUmAmGmGmCmCmCmAmGmGmGmG/3ZEN/

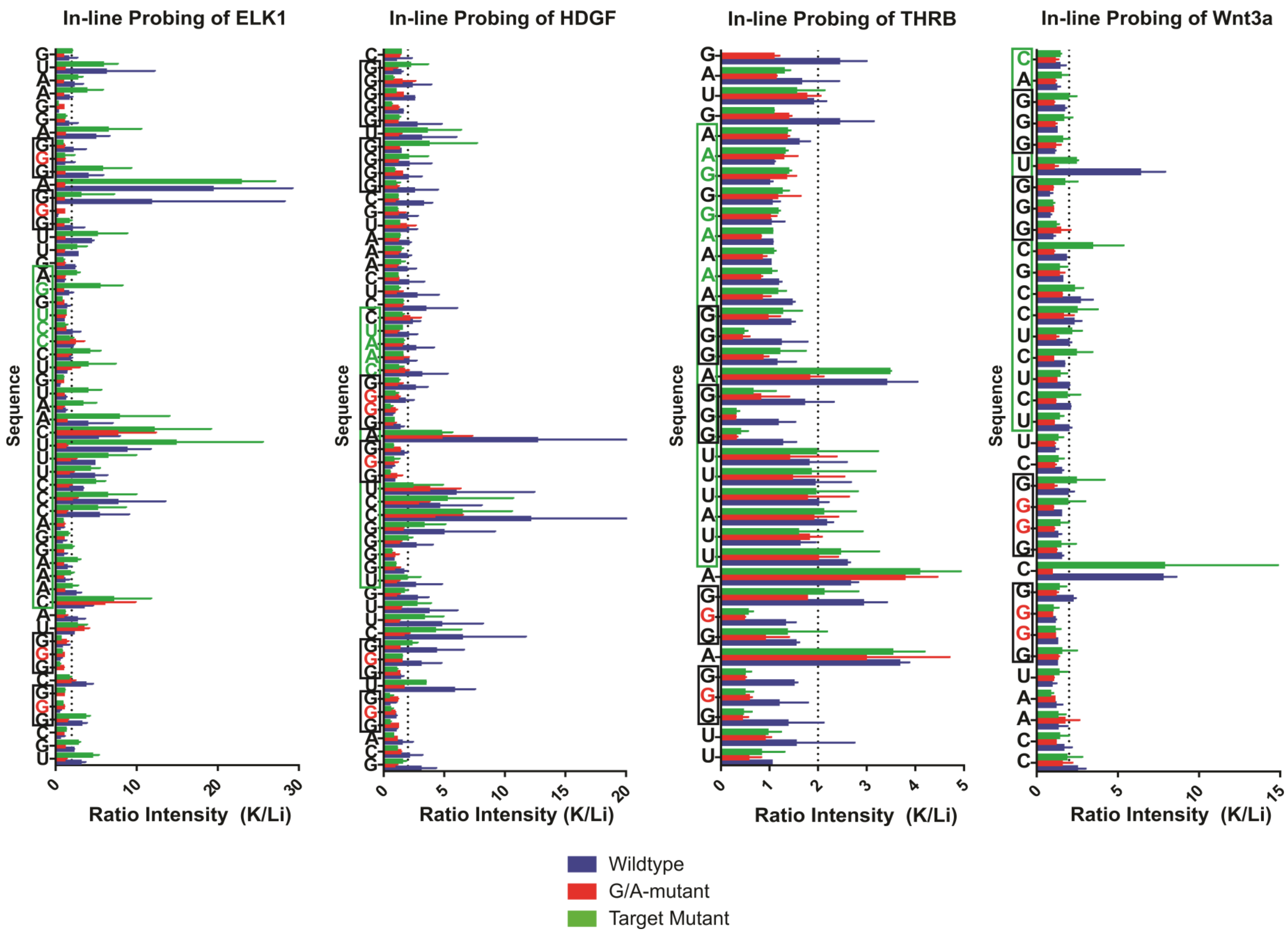
In-line Probing of ELK1
In-line Probing of HDGF
In-line Probing of THRB
In-line Probing of Wnt3a
Sequence
Ratio Intensity (K/Li)
Wildtype
G/A-mutant
Target Mutant

Supplementary Figure 1. In-line probing of ELK1, HDGF, THRB, and WNT3A.

In-line probing experiments were performed in the presence of either K^+ (favors G4) or Li^+ (does not favor G4) for the wild type, the G/A mutant and the target mutant of each gene. Each band was quantified and a K^+/Li^+ ratio was calculated for each nucleotide. A ratio >2 for the nucleotides located in the loops indicates G4 folding. The means and standard deviations were calculated from two different experiments. The guanines involved in the G4 are boxed in black and nucleotides mutated in the G/A mutants are shown in red. The miRNA binding sites are boxed in green and the nucleotides mutated in the target mutants are shown in green.

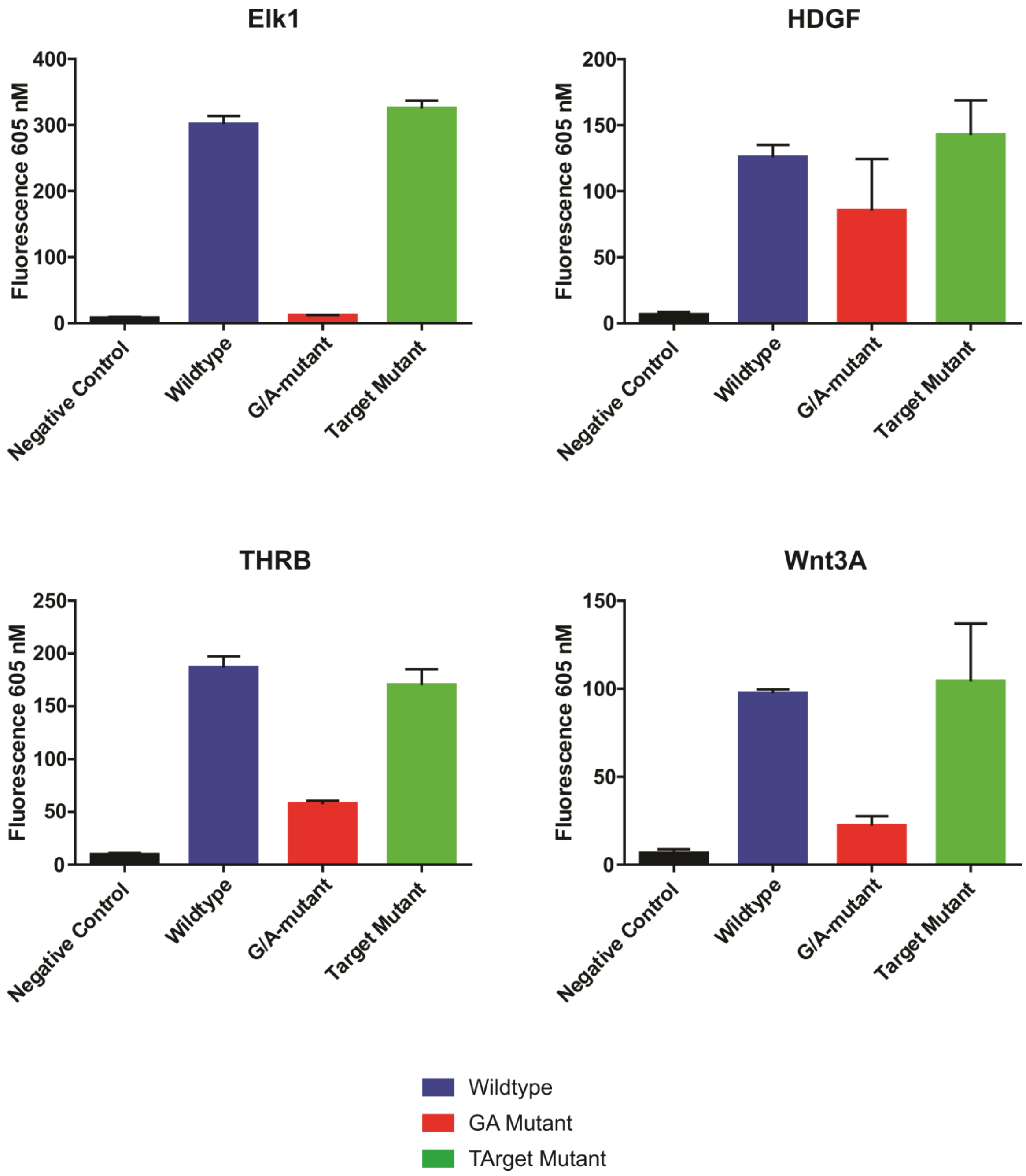

Elk1
HDGF
THRB
Wnt3A
Fluorescence 605 nM
0
100
200
300
400
0
50
100
150
200
0
50
100
150
200
250
0
50
100
150
Negative Control
Wildtype
G/A-mutant
Target Mutant
Wildtype
GA Mutant
TArget Mutant

Supplementary Figure 2. **N-methyl Mesoporphyrin IX fluorescence assay of ELK1, HDGF, THRB, and Wnt3A.**

The NMM fluorescence measured in the presence of each gene and of both its respective G/A and target mutants are shown. The means and standard deviations were calculated from two different experiments.

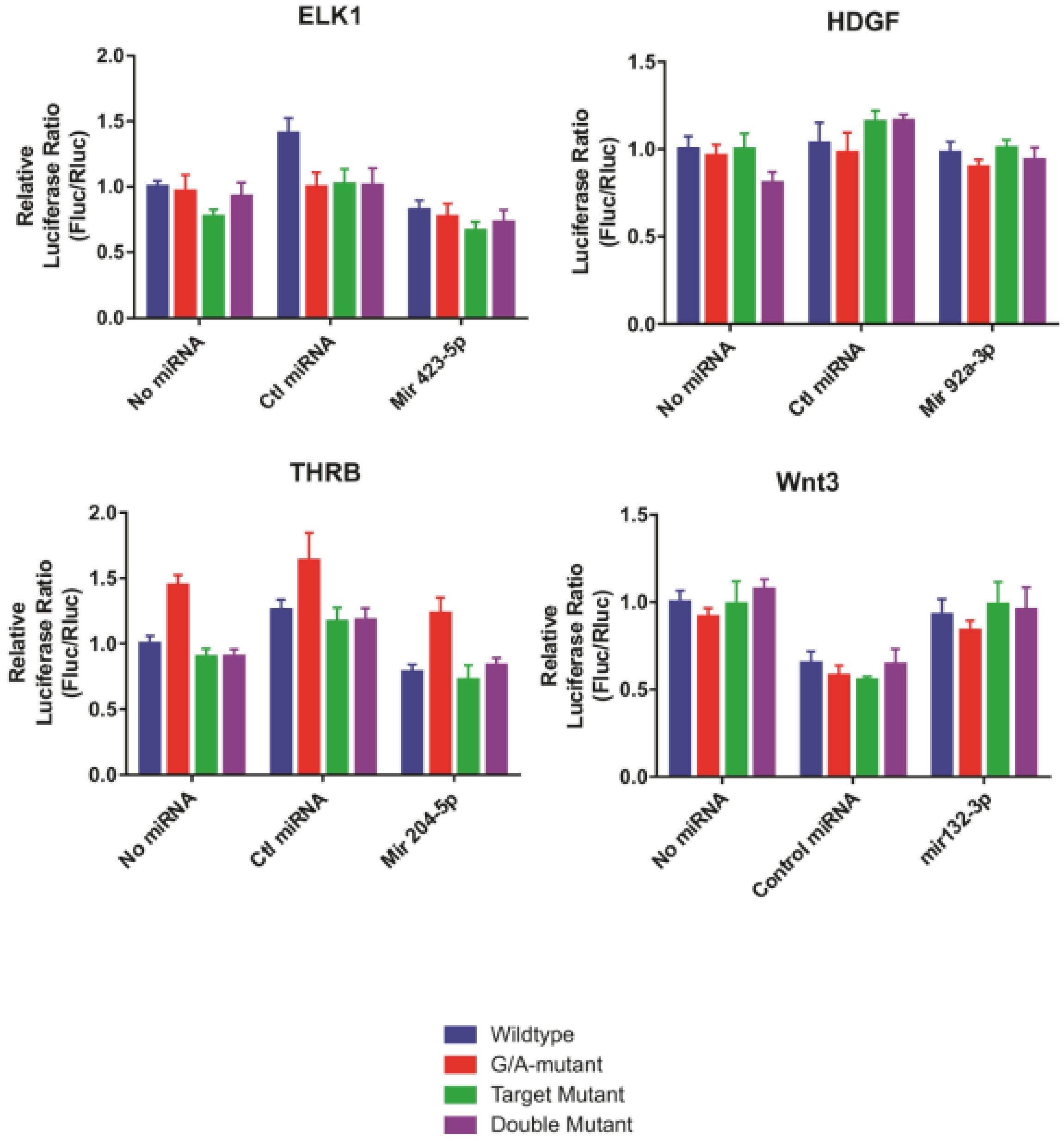

Supplementary Figure 3. **Other tested G4 do not regulate miRNA binding.**

Different ELK1, HDGF, THRB, and Wnt3A constructs (Wild type, G/A mutant, Target mutant, and Double mutant) were transfected into either HEK 293 (ELK1 THRB), Huh7 (HDGF), or HCT116

(WNT3A) cells either alone, with a control synthetic miRNA or the appropriate synthetic miRNA and the luciferase activity were measured.

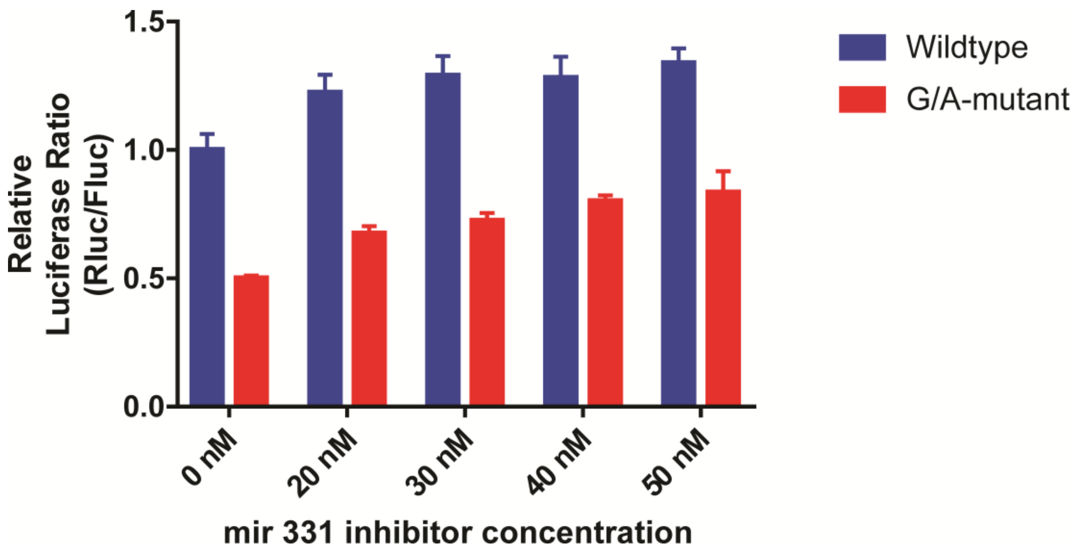

Supplementary Figure 4. Effect of a mir331 inhibitor on FADS2 reporter expression. Various concentrations of Mir331 inhibitor were transfected into HEK 293 cells. On the following day, FADS2 wild type and GA reporter luciferase were transfected. Luciferase activity was assessed on the day after the second transfection.
